# Bibliometric Evidence for a Hierarchy of the Sciences

**DOI:** 10.1371/journal.pone.0066938

**Published:** 2013-06-26

**Authors:** Daniele Fanelli, Wolfgang Glänzel

**Affiliations:** 1 Science, Technology and Innovation Studies, the University of Edinburgh, Edinburgh, United Kingdom; 2 Centre for R&D Monitoring (ECOOM), KU Leuven, Leuven, Belgium; 3 Department of Science Policy & Scientometrics, Library of the Hungarian Academy of Sciences, Budapest, Hungary; Université de Montréal, Canada

## Abstract

The hypothesis of a Hierarchy of the Sciences, first formulated in the 19^th^ century, predicts that, moving from simple and general phenomena (e.g. particle dynamics) to complex and particular (e.g. human behaviour), researchers lose ability to reach theoretical and methodological consensus. This hypothesis places each field of research along a continuum of complexity and “softness”, with profound implications for our understanding of scientific knowledge. Today, however, the idea is still unproven and philosophically overlooked, too often confused with simplistic dichotomies that contrast natural and social sciences, or science and the humanities. Empirical tests of the hypothesis have usually compared few fields and this, combined with other limitations, makes their results contradictory and inconclusive. We verified whether discipline characteristics reflect a hierarchy, a dichotomy or neither, by sampling nearly 29,000 papers published contemporaneously in 12 disciplines and measuring a set of parameters hypothesised to reflect theoretical and methodological consensus. The biological sciences had in most cases intermediate values between the physical and the social, with bio-molecular disciplines appearing harder than zoology, botany or ecology. In multivariable analyses, most of these parameters were independent predictors of the hierarchy, even when mathematics and the humanities were included. These results support a “gradualist” view of scientific knowledge, suggesting that the Hierarchy of the Sciences provides the best rational framework to understand disciplines' diversity. A deeper grasp of the relationship between subject matter's complexity and consensus could have profound implications for how we interpret, publish, popularize and administer scientific research.

## Introduction

Positivist philosopher Auguste Comte (1798–1857) first proposed a “natural” ordering of scientific disciplines based on generality of subject matter [1], [2]. From mathematics to sociology, his Hierarchy of the Sciences (HOS) was intended to reflect the growing complexity, inter-dependence, and vicinity to human passions of research fields, all of which determined their level of development as sciences. This idea was abandoned by post-positivist thinking, who increasingly emphasised the irrational side of scientific progress [3], [4], leading to the extreme opposite view that disciplines are an unordered product of historical and cultural contingencies, similar to political or artistic currents [5]. Today, concepts like “hard” and “soft” science are used in a vague, confused sense, and their imputation to specific research fields is felt to be controversial if not offensive. This might be a costly mistake, because these concepts seem to capture an essential feature of science, and have important implications that today tend to be ignored.

What do we mean by “hard” science? Scholars have treated the topic from a multitude of angles (see [6], [7], [8]), but all definitions seem to converge on the concept of consensus – consensus, for example, “on the significance of new knowledge and the continuing relevance of old” [9], [10], [11], [12]. In an ideal science, scholars share a common background of established theories, facts and methods. This allows them to agree (usually after debate and further evidence) on the validity and significance of a new research finding, making it the basis for further theorizing and research. Harder sciences are hypothesised to come closer to this ideal. Moving towards “softer” fields, this consensus becomes less likely to be reached, the common background shrinks and fractures, and so data become less able to “speak for themselves” [6]. Already in Comte's intuition, this happened primarily because of the increasing complexity of subject matters.

What do we mean by complexity? The exact definition is still debated in complexity science itself, and so are its possible measures [13], [14]. In very general terms, however, the complexity of a system is linked to the number of elements involved, their diversity, the number and non-linearity of interactions between them, the cohesiveness of internal versus external relationships (which determines how isolated the system is), the distance from thermodynamic equilibrium [15]. Complex systems require longer (uncompressible) descriptions and are less predictable in their behaviour [16], [17]. Clearly, the systems studied by individual disciplines vary widely in these characteristics. It is also clear, however, that complexity generally increases with increasing levels of organization of matter. From subatomic particles to human societies, there is an overall increase in the possible number of elements, combinations, interactions etc. the phenomenon of emergence might bring relative simplicity at higher levels, but the overall trend is for complexity to increase [16], [17]. And whilst phenomena get more complex, our ability to study them decreases. Objects of investigation become more difficult to isolate and describe, and are more diversified in space and time (e.g. [18], [19], [20]). Due to technical, practical and ethical considerations, experiments and predictions are replaced by observations and accommodations, which are arguably a less powerful and reliable sources of knowledge [21], [22], [23], [24]. These limits make replication less likely to be attempted and to be successful [25]. Moreover, the growing diversity and contingency of studied phenomena leads to a dispersion of research funding and efforts, further reducing the potential to reach conclusive evidence and settle intellectual debates (see e.g. [20]).

The fundamental prediction made by a modern version of the HOS, therefore, is that the ability of a scientific field to achieve consensus and accumulate knowledge will decrease when moving from the physical, to the biological, to the social sciences. The same prediction should hold, of course, at finer levels of analysis, and we would expect that, within each domain, individual disciplines, fields and subfields vary significantly in their level of softness. However, since we lack objective methods to measure and rank complexity at such levels, finer-grained tests would be inaccurate. How mathematics and the humanities fit into the HOS is rather unclear. In Comte's scheme, the humanities were excluded whilst mathematics was the basis of the hierarchy. The predictions developed here, however, are based on the assumption that disciplinary practices are constrained by physical properties of subject matter. Both mathematics and the humanities have purely intellectual subject matters, and therefore technically lack any physical constraint. On the other hand, these two disciplines are arguably at the extremes of a spectrum of consensus-reaching potential, and will therefore be included in a secondary test.

The HOS prediction stands in contrast with two alternatives philosophical positions. The first, very common amongst academics as well as lay-people, draws a fundamental distinction between natural and social sciences (i.e. sociology, psychology, etc.), and uses the term “soft science” to indicate the latter. Alternatively, the term “soft” is used to distinguish qualitative research or historical-philosophical studies (e.g. [26], [27], [28]). Reflecting a more general divide in Western culture [29], [30], this dichotomy can be traced back to another long-standing debate, between those who see the study of human behaviour akin to any other science, i.e. aimed at discovering general patterns and laws, and those who believe it should focus on individuality and on the meaning that people ascribe to their world and actions [31]. In principle, of course, the two purposes are not mutually exclusive, but many scholars in the social sciences and humanities maintain that consciousness, free will and socio-cultural life make human beings a completely different subject matter from those of the natural sciences [32], [33], [34], [35], [36]. The second alternative hypothesis, also very common in academia and popular culture, denies any order at all. Disciplines, under this view, deal with different phenomena and produce different kinds of knowledge, so they cannot be compared in any meaningful way – let alone be ranked (see [6], [37]). In its more radical forms, this view explicitly denies the existence of a hierarchy, and replaces it by a “disordered” view of knowledge, in which the sciences only superficially resemble each other (e.g. [38], [39]).

**Figure 1:**
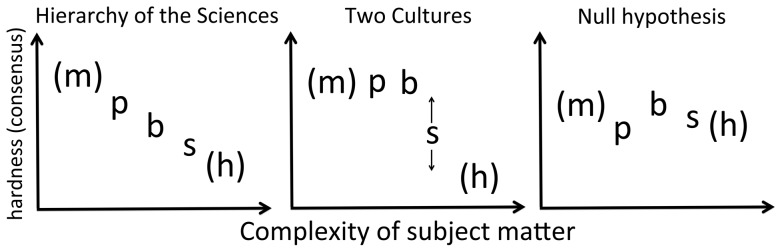
Alternative predictions tested in this study, about how scientific domains should differ in any measure of hardness (or, equivalently, consensus. m =  mathematics; p  =  physical sciences; b  =  biological sc.; s  =  social sc.; h  =  humanities). Predictions are explicit about empirical sciences, whilst mathematics and the humanities are tested secondarily.

The HOS hypothesis can therefore be readily contrasted with, on the one hand, a dichotomy hypothesis (which we will call “two cultures”) and, on the other hand, a null hypothesis, in which there is no particular order. Key to distinguishing these predictions are the biological sciences, which should fall in-between the physical and the social only according to the HOS (Figure 1).

Innumerable studies have proposed and applied measures of hardness and consensus, and reviewing them all would be beyond the scope of this paper (see [6], [7], [8]). These studies reached different conclusions, and so did their reviews. Analyses on peer review agreement on National Science Foundation grant applications, together with other evidence, led Cole (1983) to conclude that the HOS is at least partly a myth. Hard disciplines like physics, Cole argued, do appear to have a larger and more solid “core” of knowledge, manifest in the consistency of university textbooks; but at the research “front”, where science is actually done, consensus is equally low for all disciplines [10], [11], [40]. A later quantitative review, however, combined these and other empirical results and found evidence of a straightforward hierarchy [12], [41]. These contradictions are largely a consequence of methodological limitations, many of which were noted long ago [42]: most empirical studies to date have compared only one or two natural sciences (e.g. physics or molecular biology) with one or two social (usually sociology or psychology); instead of drawing representative samples, these studies focused on a few journals or specific subfields, choosing different ones every time; moreover, sample sizes in these studies are usually small, and there are remarkably few purely “null” results in the literature, which could suggest the presence of publication bias [43]. Narrative and quantitative reviews suffer form the limitations of their primary evidence, which makes their results inconclusive in turn.

Methodological biases can be avoided by using objective measures, and sociological studies of science are advancing rapidly thanks to the availability of ever more refined bibliometric data [44]. Recent studies claimed to have captured differences in consensus by looking at characteristics and networks of references [45], [46], [47], [48]. These studies, however, suffer from the confusion and limitations mentioned above, leaving the HOS and its alternatives inconclusively tested. Large cross-disciplinary studies, on the other hand, have repeatedly observed a correlation between the prevalence and growth of publication bias and putative softness, at least in non-applied research [6], [49], [50]. This suggests that there is something fundamentally true about the HOS hypothesis, with potentially important implications for how we view, publish and manage science.

This study aimed at assessing conclusively whether measurable characteristics of papers differ in ways predicted by the HOS or competing hypotheses. We sampled nearly 29,000 papers published at the beginning of 2012 in journals that Thomson Reuters' Essential Science Indicators (ESI) database classified in 12 non-applied disciplines, and measured a set of objective parameters that, in previous independent studies, had been theoretically connected to consensus and/or that had been shown to distinguish the social from the natural sciences. These parameters are: number of authors, length of article, number of cited references, proportion of books in references, age of references, diversity of cited sources, relative title length, use of first person in abstracts and likelihood to share references with other papers in the sample. Table 1 summarizes predictions for each of these parameters, whilst further explanations and details on measurement are given in the Methods section.

**Table 1:** Summary of predictions.

parameter		pred.	effects of higher consensus:	Key refs.
number of authors		+	greater scope and need for collaboration	[9], [44]
nength of article		−	less need to introduce, justify and explain study	[47], [52]
number of references		−	less need to justify, explain and support study	[47]
references to monographs		−	focus on simpler questions; less need to justify, explain and support study	[53], [54]
age of references		−	faster settling of disagreements; greater potential to build research upon previous findings	[44], [56]
diversity of sources		−	fewer research topics, which are of more general interest	[47], [57]
relative title length		+	clearly defined, substantive research questions	[52], [58]
use of first person (singular vs. plural)		−	universal validity of claims; less scope for argumentation; fewer appeals to opinion and authority	[59]
sharing of references	degree	−	clustering of studies around clearly defined, separate questions; less need to cite older and general literature	[45], [47], [48]
	intensity	+		

Predicted correlation of each bibliometric parameter with a field's level of scholarly consensus brief explanation of hypothesised causal mechanism, and studies from which the predictions were derived. Extensive explanations for each parameter are given in the Methods section.

In what we define as the “main test”, we assessed how the biological sciences compare to the physical and the social – the basic prediction being that the former should have intermediate characteristics between the latter two. To make this test more balanced and powerful, we sub-grouped the four ESI biological disciplines in two harder and two softer, under the prediction that the latter should fall between the former and the social sciences. We also run an “extended test”, to assess whether mathematics and the humanities match intuitive predictions, and finally present data disaggregated by ESI discipline.

## Methods

### Sampling

We sampled research articles from journals covered by Thomson Reuters' Web of Science database, assigned by the Essential Science Indicators scheme to the categories of: Mathematics, Space Science, Physics, Chemistry, Molecular Biology, Biology & Biochemistry, Plant and Animal Sciences, Environment/Ecology, Psychiatry/Psychology, Economics & Business, Social Sciences General, and Humanities (this latter identified with journals listed in the Arts & Humanities Citation Index). These categories cover basic (non-applied) research, which is where predictions of the HOS apply [1], [6]. To truly capture the research frontier, we selected the “first generation” of papers published in 2012 (i.e. published in their journal's first 2012 issue and/or in January), obtaining a final sample of 28,893 papers, of which 28,477 had a non-empty references list – covering over 1,140,000 references in total. Based on theory and a preliminary study [51] we focused on the following measures.

#### Number of authors

Research teams are almost by definition built around a consensus on objectives and methods. Moreover, the ability to study a problem with greater accuracy and detail leads to a specialization of roles, making collaboration essential [9]. The hardness of a field, therefore, should be manifest in the size of its research teams. Alternative arguments would suggest that team size is a consequence of funding availability, rather than consensus *per se* [44].

#### Length of article

When consensus is lower, papers must put greater efforts in describing the background, justify their rationale and approach, back up their claims and extensively discuss their findings [47], [52]. Longer introductions, and generally longer papers, should therefore characterize softer research. We measured the total number of pages.

#### Number of references

For reasons similar to those that make an article longer, references to previous literature should also be more numerous in low-consensus fields [47].

#### References to monographs

Scholars in the humanities and social sciences still frequently choose to publish books rather than papers. This could be the effect of tradition, or of the greater amount of space needed to analyse complex phenomena. Previous studies have observed higher citations to monographs in the social sciences, and intermediate values in at least some biological disciplines [53], [54]. These studies classified references using rules of thumb, whose error rate can be quite high [54]. To obtain a more precise measure, we combined these rules of thumb with automatic searches in Google-Books and in text lists of journal titles. Searches in Google-Books that returned valid results were classified as monographs. Source titles that matched journal lists, and references that included volume and page number were classified as journal articles, the rest was hand-classified as either of the above or as “other” (a category that included conference proceedings and thesis, but which was then not actually used in these analyses). Uncertain attributions (lacking most information and having ambiguous titles) were classified as monographs – therefore, the data presented here are an upper estimate of the proportion of books. However, classifying uncertain items as any other category yielded substantially similar results.

#### Age of references

Having noted that some sciences “metabolize” the literature more rapidly, Derek de Solla Price proposed an index, which measures the proportion of cited references published in the five years preceding the citing paper [44]. This index was repeatedly shown to distinguish the social and natural sciences, and is therefore considered *the* measure of hardness (e.g. [55]). Much attention is still paid to this parameter (e.g. [56]), yet no study assessed how accurately it reflects the HOS. In a preliminary study, we compared this index to other measures [51], and Price's index emerged as equally powerful and computationally simpler. For monographs, which tend to be cited in their later editions, we used the oldest year returned by Google-Books search (see above).

#### Diversity of sources

When scholars agree on the relative importance of scientific problems, their efforts will concentrate in specific fields and their findings will be of more general interest, leading to a greater concentration of the relevant literature in few, high-ranking outlets. From harder to softer fields, therefore, we predict a growing diversity of sources of information [47]. Our preliminary study tried different diversity measures – concluding in favour of Shannon's diversity index, in agreement with independent studies [57]. We also tried limiting this measure to journals alone, finding little substantial differences in the results. Here we measured the Shannon diversity of all cited sources (titles of journals, conferences, books, etc.…)

#### Relative title length

Linguistic analyses of scientific papers noted that the number of substantive words in titles tended to be longer and to correlate with an article's total length in harder fields [52], [58]. This was interpreted as reflecting the greater empirical focus and efficiency of high-consensus fields, but the evidence was deemed too limited to draw firm conclusions. We measured the total number of words, divided by total number of pages.

#### Use of first person

**Figure 2:**
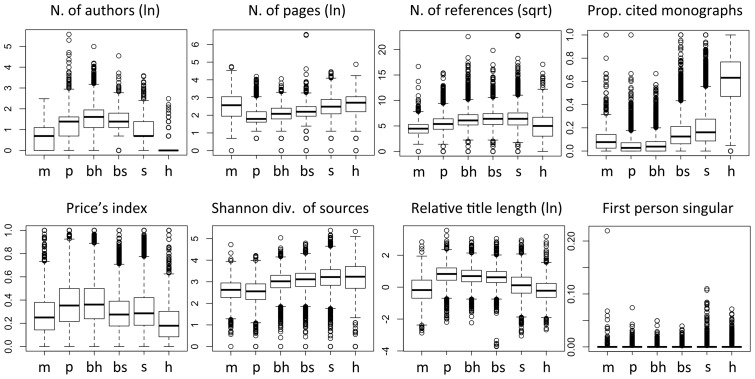
Paper characteristics hypothesised to reflect the level of consensus, by scientific domain. Domains are attributed based on journal, following the classifications of Essential Science Indicators and Arts & Humanities Science Citation Index: m  =  mathematics; p  =  physical sciences (Space Sc.+ Physics + Chemistry); bh  =  biological-hard sciences (Molecular Biology + Biology & Biochemistry); bs  =  biological-soft sciences (Plant and Animal Sc. + Environment/Ecology); s  =  social sciences (Psychiatry/Psychology + Economics & Business + Social Sciences, General); h  =  humanities. [Data sourced from Thomson Reuters Web of Knowledge].

Scientists aim at making universal claims, and their style of writing tends to be as impersonal as possible. In the humanities, on the other hand, the emphasis tends to be on originality, individuality and argumentation, which makes the use of first person more common [59]. We therefore hypothesised that the hierarchy of the sciences could reflect the frequency of use of personal pronouns. Of all parameters in the study, this is the one less directly linked to consensus and complexity of subject matter, and more likely to be determined by tradition, disciplinary convention, or journal style recommendations. We calculated the proportion of first person pronouns, both singular and plural (i.e. “I”, “me”, “mine”, “we”, “our” etc.) among all words in the abstract. Our main prediction was that authors would under-use personal pronouns in harder sciences. We created a dummy variable separating single- and multi-authored papers, giving them values of -1 and 1, respectively. Greater use of singular pronouns in single-authored papers would be revealed by a negative value of the interaction term, whilst greater use of plural pronouns by a positive one. In the figures (e.g. Figure 2) we give prominence to the use of first person, due to limitations of space.

#### Sharing of references

Authors that cite a common literature almost by definition are exhibiting a common cognitive background. The sharing of references between papers, therefore, is perhaps the most direct expression of scholarly consensus. Of the various techniques available to analyse citation networks, the most likely to reflect this parameter is bibliographic coupling, in which a network link is draw between two papers that cite the same reference [46], [60], [61].

Recent studies using this approach suggested that: 1-papers published in special issues in the natural sciences had more references in common, whilst the social sciences shared older references [47]; 2-shared references are more unequally distributed in physics compared to psychology [45]; 3-citation networks of biophysics show greater coherence and less semantic fragmentation than in economics and sociology [48]. In addition to general limitations noted in the Introduction to this paper, most bibliographic coupling studies sampled papers across multiple years. Doing this might introduce a confounding factor, because earlier papers might inspire themes and references to authors of later papers. True scholarly consensus is maximally expressed when two scholars cite the same literature without knowing of each other's work. To try to capture this effect, we sampled papers that were published almost simultaneously (i.e. January 2012 and/or first issue of the year).

We determined whether any two papers in the sample shared one or more cited references, independent of discipline. To reduce errors, references were initially compared using the DOI number and, if this was unavailable, by using a string that included author, year and source (i.e. ignoring volume and page information, which are less reliable). Harder sciences are expected to share more references, at least amongst the recent literature, but also to show a greater focus on several specific problems, leading to an overall greater clustering of the network. In other words, they are predicted to share a greater number of references with fewer other papers.

Harder sciences would also be predicted to share more recent literature [47]. We initially attempted to partition the network by age of references (i.e. above and below median age in each paper), finding a greater sharing of older literature in the social sciences, as expected. However, since disciplines differ in the average age of cited references, what was classified as “old” in one paper was sometimes classified as “new” in another, making this operation dubious. Therefore, we chose not to partition references by age, and limited analyses to the overall number of connections between papers (i.e. node degree, which we will call “sharing degree”, or “degree” for brevity), the number of references shared between each (i.e. weight of edges, which we averaged across all edges of a node obtaining what we call “sharing intensity”), and overall structure characteristics of the network (i.e. density, modularity etc.).

### Statistical analyses

For each bibliographic parameter, we tested predictions twice: once excluding and once including mathematics (we will call these “basic” and “extended” tests). To make the tests more accurate, we split the biological sciences between two ESI categories that would be predicted, by the HOS, to be harder (i.e. molecular biology, and biology and biochemistry) and two softer (i.e. plant and animal sciences, and environment/ecology).

The likelihood of papers in each domain to share references was measured with Exponential Random Graph Modelling, which estimates the probability of a network configuration as a whole [62], [63]. ERGMs are the only technically correct way to test for node-level predictors, because they take into account the non-independence of links and other possible confounding factors. Unfortunately, current algorithms are unable to incorporate information on the weight of links (which in our case would reflect the number of references shared between two papers). To test for this latter factor, we separated the network in subsets based on edge weight (from 1 to ≥10), and analysed the distribution of edges separately. In each case, we assessed the likelihood of a node (i.e. paper) to form a link of weight w (i.e. to share w references with another paper) depending on its domain, and controlling for each node's number of references, overall number of edges and for presence of triangle-effects (if paper x shares publications with y and with z, then these latter are more likely to share references with each other). The ERGM, estimated by maximum-pseudo-likelihood, was specified as follows:
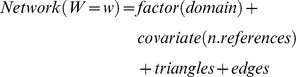
Where W is edge weight (w∈{1,2,3,4,5,6,7,8,9,>9}), domain and references are attributes of nodes (i.e. of papers), and triangles and edges are attributes of the network.

**Figure 3:**
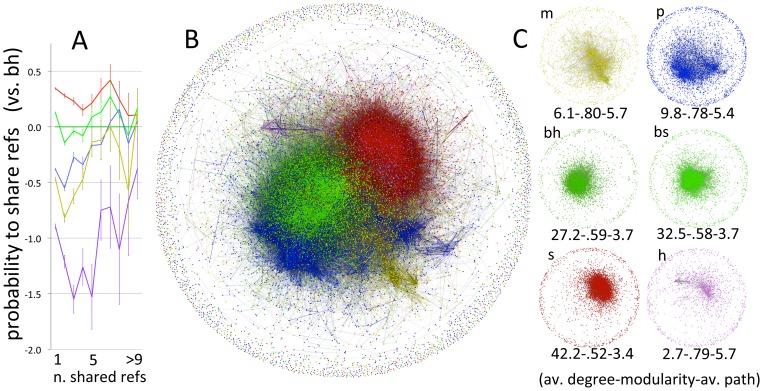
Bibliographic coupling network of papers, partitioned by scientific domain (total N = 28,477; yellow  =  mathematics; blue  =  physical sciences; darker green  =  biological-hard sc.; lighter green  =  biological-soft sc.; red  =  social sc.; purple  =  humanities). Panel A: probabilities to share a given number of references with any other paper in the sample, estimated by exponential random graph modelling. The model controlled for number of references cited by each paper, number of triangles and edges. Error bars are 95% Confidence Intervals, bh is the reference category, and has therefore all values set to zero. Panel B: network of shared references, in Yifan Hu Proportional layout. Panel C: network partitioned by domain, with average degree, modularity and average path length. [Data sourced from Thomson Reuters Web of Knowledge].

These analyses yield probability estimates and standard errors analogous to those of a logistic regression, which were used to produce the values and confidence intervals plotted in Figure 3A.

Regression analyses used a generalised linear model. Each bibliographic parameter was assessed for its distribution, and was either transformed to approach normality or, whenever possible, tested untransformed, by adopting an appropriate link function. Both the main and extended test included possible confounders as independent co-variables whenever required, as specified in the text. The general linear form of these regression models was specified, for all parameters except the use of singular pronoun, as:
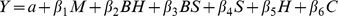
where Y is the parameter of interest, α is the intercept, C is an eventual confounding variable and the remaining factors are scientific domains (abbreviations as in figures), with physical sciences as reference category. Confounding variables and eventual weighting were added in some analyses, as specified in the text.

The use of singular pronoun was assessed in its interaction term with a dummy variable X, in a hierarchically well-formulated model (i.e. a model in which all lower-order terms of an interaction are included [64]).
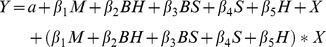
Where X = −1 for single-authored papers, and X = 1 for multiple authored papers. The model was weighted by total number of words in the abstract. Only the values of interaction terms are reported in the text.

Multivariable analyses followed a reverse logic, and used ordinal regression to test the ability of each parameter to predict the order of disciplines. This method, technical similar to a logistic regression, was chosen for its robustness and limited data assumptions. We specified a generalized linear model with logit link-function, measuring the probability to observe a rank, given the predictors:
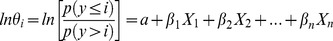
where i is the rank of scientific domain (i.e. m<p<bs<bh<s<h) and X_n_ are bibliometric parameters as specified in the text.

**Figure 4:**
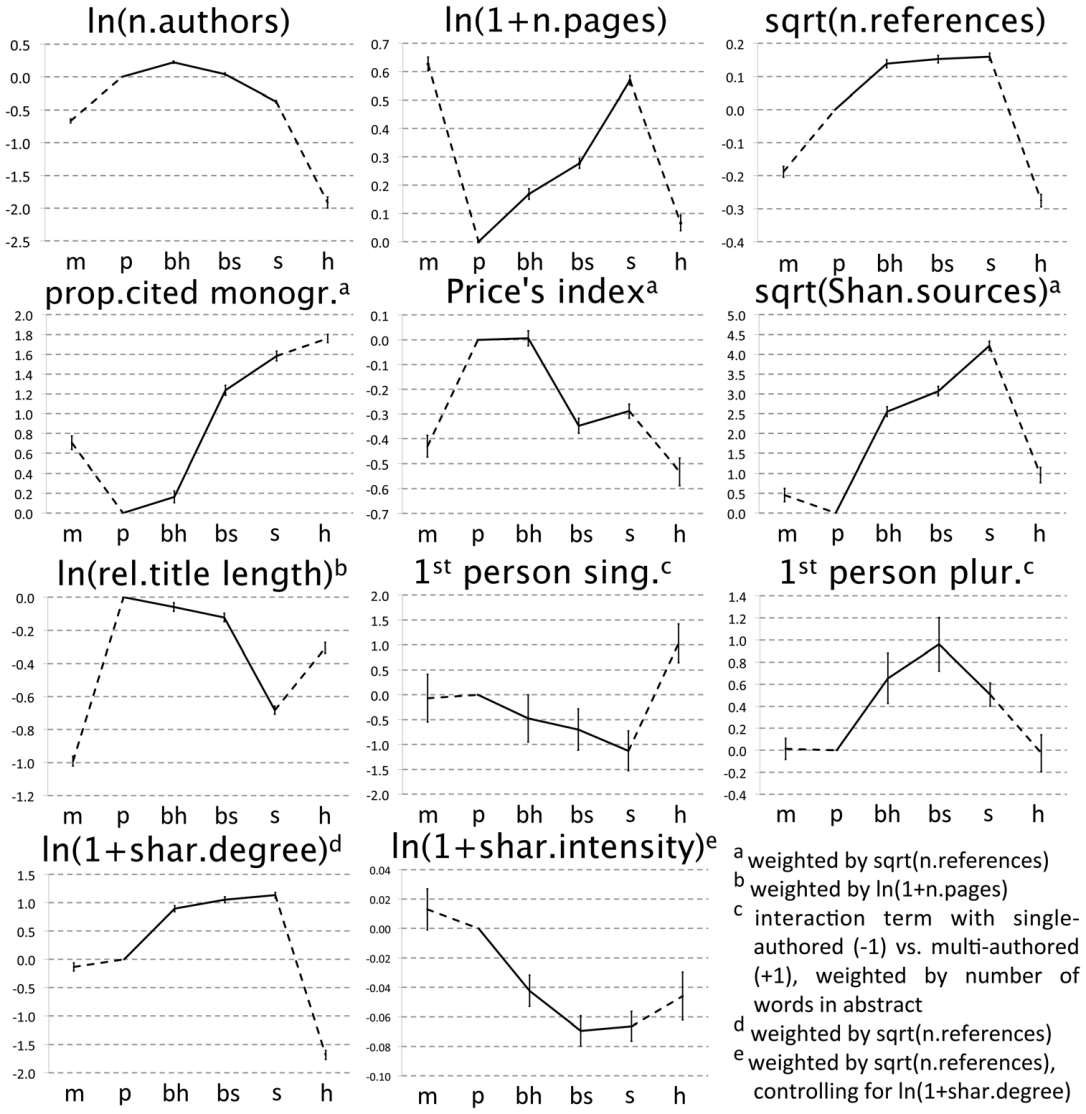
Estimates of regression analyses with, as dependent variable parameters hypothesised to reflect consensus and, as independent variables, scientific domains, weighted or corrected as described. Bars are 95% confidence intervals, and lines are added to help visualize trends, with solid and dotted lines representing, respectively, main and extended test. Physical sciences are the reference category, and therefore have values set to zero. m = mathematics; bh =  hard-biological disciplines (Molecular Biology + Biology & Biochemistry); bs =  soft-biological disciplines (Plant and Animal Sciences + Environment/Ecology); s =  social sciences (Psychiatry/Psychology+Economics & Business+Social Sciences, general), h = humanities. See methods for further details on Methods section, and for the exact regression results with standard error see Table S1. [Data sourced from Thomson Reuters Web of Knowledge].

Network node data, i.e. the degree and intensity of sharing, was included in regression models, treating values for each node as independent. This obviously violates assumptions of independence, a violation that leaves the magnitude and direction of effects unaltered, but might lead to an underestimation of standard errors – and therefore of statistical significance. Statistical significance, however, is hardly an issue in this study, because the statistical power is very high even for small effects. In a univariate regression model, for example, we have over 99.9% statistical power to detect effects of Cohen's f^2^ as small as 0.001 [65]. This power can be appreciated in Figure 4, where the 95% confidence intervals (all calculated as 1.96*standard error of mean) are extremely narrow for most effects. What is really relevant in these analyses, in other words, is not whether they pass a formal 0.05 statistical significance threshold, but whether these parameters place disciplines in the predicted order, and how strong each effect is. In any case, we assessed the robustness of results by excluding network parameters.

All statistical analyses were performed with basic and specific libraries from the open source package R [66], [67], [68]. Statistical power was estimated with G*Power v. 3.1 [69]. The bibliographic coupling network was created with purposely written Java code, and network images and statistics were produced with the open source software Gephi (v. 0.8 alpha) [70].

## Results

With three minor exceptions, all bibliometric parameters placed the biological sciences between the physical and the social, and placed the biological-hard sciences before the biological-soft (Figure 2). The mean number of authors peaked in the hard-biological sciences, although the extreme values of this parameter – in other words, the largest collaborations of all – followed the trend predicted by the HOS. The use of first person showed some discontinuity between the natural and social sciences for the singular form, whilst for the plural form it showed greater similarities between the biological and the social sciences than would be expected under any hypothesis (Figure S1).

Bibliographic coupling parameters also generally supported the HOS (Figure 3). The likelihood to share references with any other paper in the network had intermediate values for the biological sciences, both hard and soft. Moreover, whilst the physical sciences were likely to share many references with fewer other papers, the opposite was true for the social sciences (Figure 3A). This was reflected in the structure of the network (Figure 3B), harder domains having lower average degrees, lower density and greater modularity, and softer ones progressively losing coherence (Figure 3C). The pattern was only broken by the humanities (Figure 3B, C).

Multiple regression analysis on individual parameters, which controlled for possible confounding factors, generally confirmed the above observations. All parameters placed the biological-hard sciences between the physical and the biological-soft, and these latter before the social sciences or on a par with them, except for the number of authors and for the use of first person plural (Figure 4 and Table S1).

When tested together in a multiple ordinal regression model, the main test was strongly supported: all parameters significantly predicted the HOS, mostly with large effects (Table 2). Only exception was the number of references, which had opposite sign to what was predicted. This parameter, however, showed very high colinearity (which was unsurprising, since most parameters in the model are calculated from the reference list), and was therefore removed from main effects and retained in the model only as weighting factor. Predictions were substantially supported in the extended test, too, although the magnitude of some effects was reduced, and one parameter had its sign reversed (i.e. relative title length) (Table 2). Surprisingly, Price's index was amongst the weakest predictors. As noted above, assumptions of independence are violated by the network parameters. Removing these from the model, however, did not change the results in any substantial way.

**Table 2:** Main and extended test, all parameters combined.

	main test		extended test	
predictor	b±se	z	b±se	z
ln(n. authors)	−0.433±0.010	−42.83	−0.088±0.009	−9.508
Price's index	−0.404±0.029	−13.932	−0.069±0.026	−2.655
sqrt(Shannon diversity of sources)	0.082±0.001	42.443	0.110±0.001	63.688
proportion of cited monographs	6.626±0.054	121.222	7.505±0.045	165.223
ln(1+n. pages)	0.899±0.021	41.514	0.596±0.019	30.991
ln(relative title length)	−0.118±0.015	−7.56	0.218±0.014	15.561
1^st^ pers. singular	12.23±2.333	5.245	12.32±1.568	7.854
1^st^ pers. plural	−22.21±1.182	−18.787	−67.44±0.771	−87.429
single vs. multi-author dummy	−0.096±0.013	−7.147	−0.303±0.011	−26.895
ln(1+sharing degree)	0.227±0.003	58.566	0.252±0.003	70.329
ln(1+sharing intensity)	−0.418±0.020	−20.482	−0.382±0.017	−21.786
1^st^ pers. singular *(sing vs. multi author)	−27.78±2.332	−11.912	−17.74±1.568	−11.312
1^st^ pers. plural*(sing vs. multi author)	16.61±1.179	14.088	41.14±0.757	54.34

Multiple ordinal regression assessing how parameters hypothesised to reflect consensus predict a rank order of scientific domains (main test: I-physical, II-biological-hard, III-biological-soft, IV-social sciences; extended test: same as main, with mathematics and humanities at the two ends). For details on each parameter, see introduction and methods. Use of first person pronouns was measured through an interaction term with a dummy variable separating single and multi-authored papers – the main effects for these latter two are retained to ensure a hierarchically well-formulated model. [Data sourced from Thomson Reuters Web of Knowledge].

**Figure 5:**
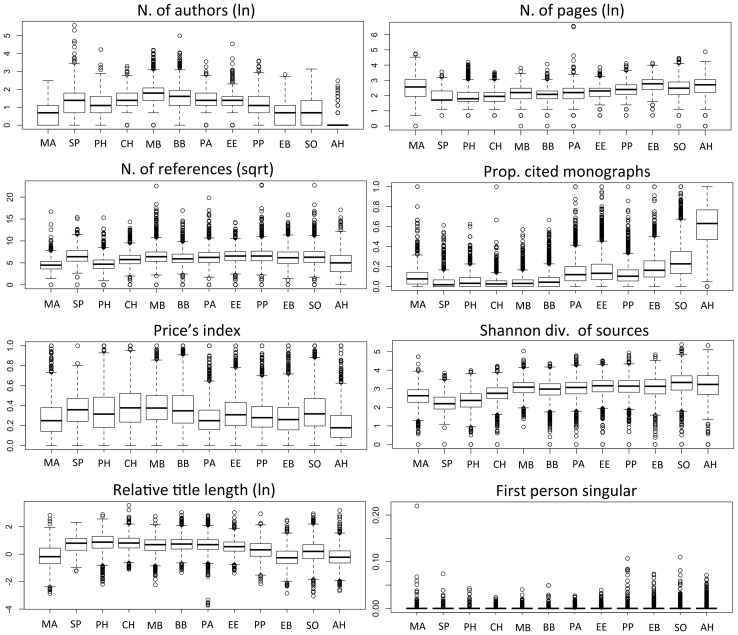
Paper characteristics hypothesised to reflect the level of consensus, by scientific discipline. Classification is based on journal, following the systems of Essential Science Indicators and Arts & Humanities Science Citation Index: ma  =  mathematics; sp  =  Space Science; ph =  Physics; ch  =  Chemistry; mb  =  Molecular Biology; bb  =  Biology & Biochemistry; pa  =  Plant and Animal Sciences; ee  =  Environment/Ecology; pp  =  Psychiatry/Psychology; eb  =  Economics & Business; so  =  Social Sciences, General; ah  =  Arts & Humanities. [Data sourced from Thomson Reuters Web of Knowledge].

**Figure 6:**
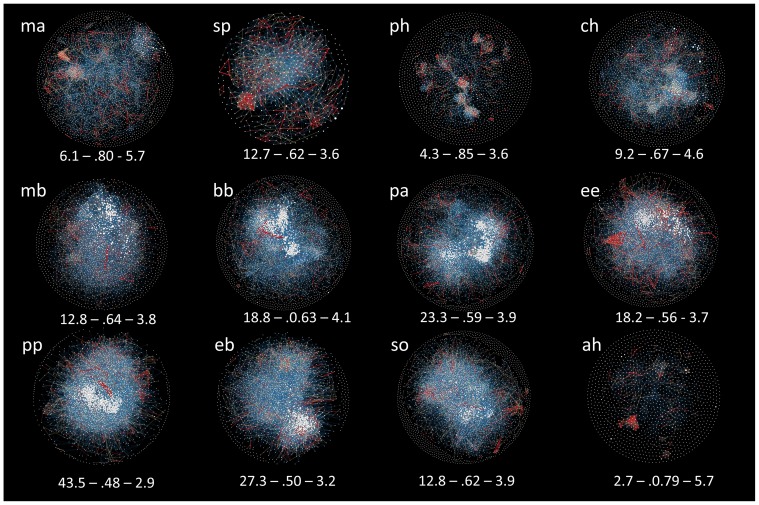
Bibliographic coupling networks, in Fruchterman-Reingold layout, with node size proportional to degree, and edge size and colour reflecting weight (i.e. number of shared references between any two papers: blue = 1; yellow =  ≥2; red ≥5). Numbers report average degree, modularity and average path length. Classification based on journal, following the systems of Essential Science Indicators and Arts & Humanities Science Citation Index: ma  =  mathematics; sp  =  Space Science; ph =  Physics; ch  =  Chemistry; mb  =  Molecular Biology; bb  =  Biology & Biochemistry; pa  =  Plant and Animal Sciences; ee  =  Environment/Ecology; pp  =  Psychiatry/Psychology; eb  =  Economics & Business; so  =  Social Sciences, general; ah  =  Arts & Humanities. High-resolution images available from the authors. [Data sourced from Thomson Reuters Web of Knowledge].

As explained in the introduction, we focused on broad domains to keep predictions objective. However, when the 12 disciplines were ordered following an intuitive notion of complexity, they still showed smooth transitions for most parameters, particularly amongst the natural sciences (Figure 5 and Figure S2). Reference-sharing networks showed the predicted gradual loss of structure moving towards the social sciences, albeit with a possible discontinuity amongst the soft-biological, and a peak in Psychiatry/Psychology rather than in Social Sciences, General (Figure 6).

## Discussion

We sampled nearly 30,000 papers from 12 disciplines and measured a set of parameters that previous studies suggested would reflect the level of scholarly consensus. In all but a few of the tests, the biological sciences had values intermediate between those of the physical and the social sciences, and putatively “softer” biological sciences fell in-between molecular-based biology and the social sciences. If a natural vs. social, or a science vs. non-science dichotomy were true, trends should have appeared discontinuous. If neither theory were true, disciplines should have been distributed randomly with respect to any characteristic – which, given the number of parameters tested, was statistically the most likely scenario. Therefore, these results strongly support the Hierarchy of the Sciences, against alternative theories of scientific knowledge.

Moving from mathematics to the humanities, or at least from the physical to the social sciences, papers progressively tend to list fewer co-authors, have longer texts, use less substantive titles, make greater use of first person pronouns, and cite more references, more books, older literature, and a higher diversity of sources. Perhaps most important of all, papers show, collectively, a proportional loss of cognitive structure and coherence in their literature background: in the physical sciences, they share several references with fewer other papers, as we expect if studies cluster around clearly defined problems and methods; moving to the biological and to the social sciences, papers are increasingly likely to share common references randomly, which reflects the greater freedom and flexibility with which scientists establish a cognitive basis to their research (Figures 3 and 5).

What exactly causes these patterns is far from understood, and we are prepared to discover that some of the assumptions behind our empirical predictions are wrong – after all, quantitative studies of science like this one are rather soft. Nonetheless, the data unequivocally point to a “gradualist” view of the sciences, which needs an explanation. A causal link between complexity of subject matter and ability of scholars to reach consensus is the best explanation we have.

Scientometric epistemological studies proceeding from predefined subject-classification schemes run a risk of circularity, which this study should have avoided. One could plausibly argue that the Web of Science classification system is partly inspired by a HOS prejudice. Categories such as “social sciences, general”, for example, could have been created around looser criteria, and therefore might include a wider variety of journals compared to traditional categories like “Space Science”. This difference could explain why we observe higher diversity and less coherence amongst social sciences' references. We believe this to be a possible limitation for comparisons at the level of disciplines, but not for broad domains, which are objective categories: the physical sciences deal with non-biological phenomena, the social sciences with human behaviour etc. Intriguingly, a previous study that examined the distribution of methodologies in ESI disciplines found greater variability amongst physical and biological disciplines, where many studies are actually behavioural [6]. So if any flaws exist in the ESI classification, they probably played against the HOS hypothesis.

Promoters of the “cultural” paradigm might still claim that we only observed differences in cultural practices. They could maintain, in particular, that researchers in, say, sociology simply “learn” to write longer papers, collaborate less, refer to older literature etc. Even more subtly, critics might argue that consensus in any particular discipline is achieved not because data “speak clearly”, but because sociological factors push researchers to adhere to one paradigm despite contrary evidence. We do not deny that disciplinary practices, including the ones measured here, have strong cultural and generally non-cognitive components. However, the most parsimonious explanation for our findings is that such culturally transmitted practices are shaped, to some extent, by objective constraints imposed by subject matter. This follows from at least two considerations. First, some of the parameters, in particular those extracted by bibliographic coupling, represent collective phenomena, which are beyond the conscious control of any individual actor. Second, as explained in the introduction, a hierarchical view of science is much less popular, nowadays, that a natural-social dichotomy: if anything, many disciplines are criticised for succumbing to “physics envy” – i.e. hopelessly striving to reach the accuracy, credibility and prestige accorded to astronomy or quantum theory [71], [72]. Therefore, if research practices were all arbitrary and culturally imposed, then we would expect most disciplines to look, superficially, like astrophysics. Interestingly, the parameter most likely to reflect just stylistic conventions – the linguistic use of first person pronouns – was the one most supportive of the two-cultures hypothesis (Figure 2, Figure 4 and Table S1). We predict that other non-cognitive parameters may also show natural-social dichotomies.

Ignoring the relationship between scientific consensus and subject matter's complexity could be a costly mistake. Theory and empirical evidence, for example, suggest that the frequency of false positives and publication biases vary with the level of scholarly consensus [6], [73], [74], [75]. This would imply that claims made in softer fields should be backed up by greater debate and replication efforts before being accepted. This fact is often forgotten by the media and policy makers, who at best discuss uncertainty on a case-by-case basis. This fact also tends to be ignored by systems of scientific publication and career advancement, which in all fields tend to reward “pioneering” findings reported for the first time in prestigious journals. Managing all sciences in the same way might be a recipe for producing more false positives, biased findings, and scientific misconduct in softer fields [6], [49], [50]. It could be more than a coincidence that physicist Jan Hendrik Schön's duplicated graphs took less than a year to be discovered, whereas social psychologist Diederik Stapel could fabricate his way through 20 years of star-level career, with no one ever challenging his work [76], [77]. Perhaps, soft sciences would progress more rapidly if their practitioners were rewarded not based on immediacy and impact, but on methodological transparency and successful replication [78], [79]. We emphasize that this argument does not refer exclusively to the social sciences or humanities. Softer fields are likely to be found, even if perhaps at lower frequencies, in the physical and the biological sciences. Conversely, there is no reason why high-consensus fields should not exist in the social sciences, too.

This study conclusively proved a general pattern, the details and the causes of which remain to be uncovered. Research should clarify, in particular, how complexity of subject matter and other field-specific factors affect research practices and scholarly consensus. Already at the level of broad discipline categories, we have made unexpected observations, with putatively harder fields exhibiting soft-like characteristics. Reference-sharing patterns in Psychiatry/Psychology or Plant and Animal Sciences, for example, would suggest less cognitive coherence than for Environment/Ecology or Economics & Business, respectively, despite the fact that these latter study higher-order phenomena (Figure 6). It is important to note, however, that the ESI classification for these four disciplines combines pure and applied research, which might represent an important confounding factor in our analyses [6]. Future progress might come from finer-grained empirical studies, which compared fields using more refined classifications or even, if at all possible, ranking the complexity of subject matters directly, independent of any disciplinary connotation.

## Supporting Information

Figure S1 **Frequency of first person plural pronouns in abstracts, by scientific domain.** Domains are attributed based on journal, following the classifications of Essential Science Indicators and Arts & Humanities Science Citation Index: m  =  mathematics; p  =  physical sciences (Space Science + Physics + Chemistry); bh  =  hard-biological disciplines (Molecular Biology + Biology & Biochemistry); bs  =  soft-biological disciplines (Plant and Animal Sciences + Environment/Ecology); s  =  social sciences (Psychiatry/Psychology + Economics & Business + Social Sciences, general); h  =  Humanities. [Data sourced from Thomson Reuters Web of Knowledge].
(CORR)

Figure S2 **Frequency of first person plural pronouns in abstracts, by scientific domain.** Domains are attributed based on journal, following the classifications of Essential Science Indicators and Arts & Humanities Science Citation Index: m  =  mathematics; p  =  physical sciences (Space Science + Physics + Chemistry); bh  =  hard-biological disciplines (Molecular Biology + Biology & Biochemistry); bs  =  soft-biological disciplines (Plant and Animal Sciences + Environment/Ecology); s  =   =  social sciences (Psychiatry/Psychology + Economics & Business + Social Sciences, general); h  =  Humanities. [Data sourced from Thomson Reuters Web of Knowledge].
(CORR)

Table S1 **Main and extended test, individual parameters.**
(DOCX)
