# Supplementary figures and images for: Bibliometric Evidence for a Hierarchy of the Sciences
Source: PLoS One. 2013 Jun 26;8(6):e66938. doi: 10.1371/journal.pone.0066938 (PMC3694152; doi:10.1371/journal.pone.0066938)

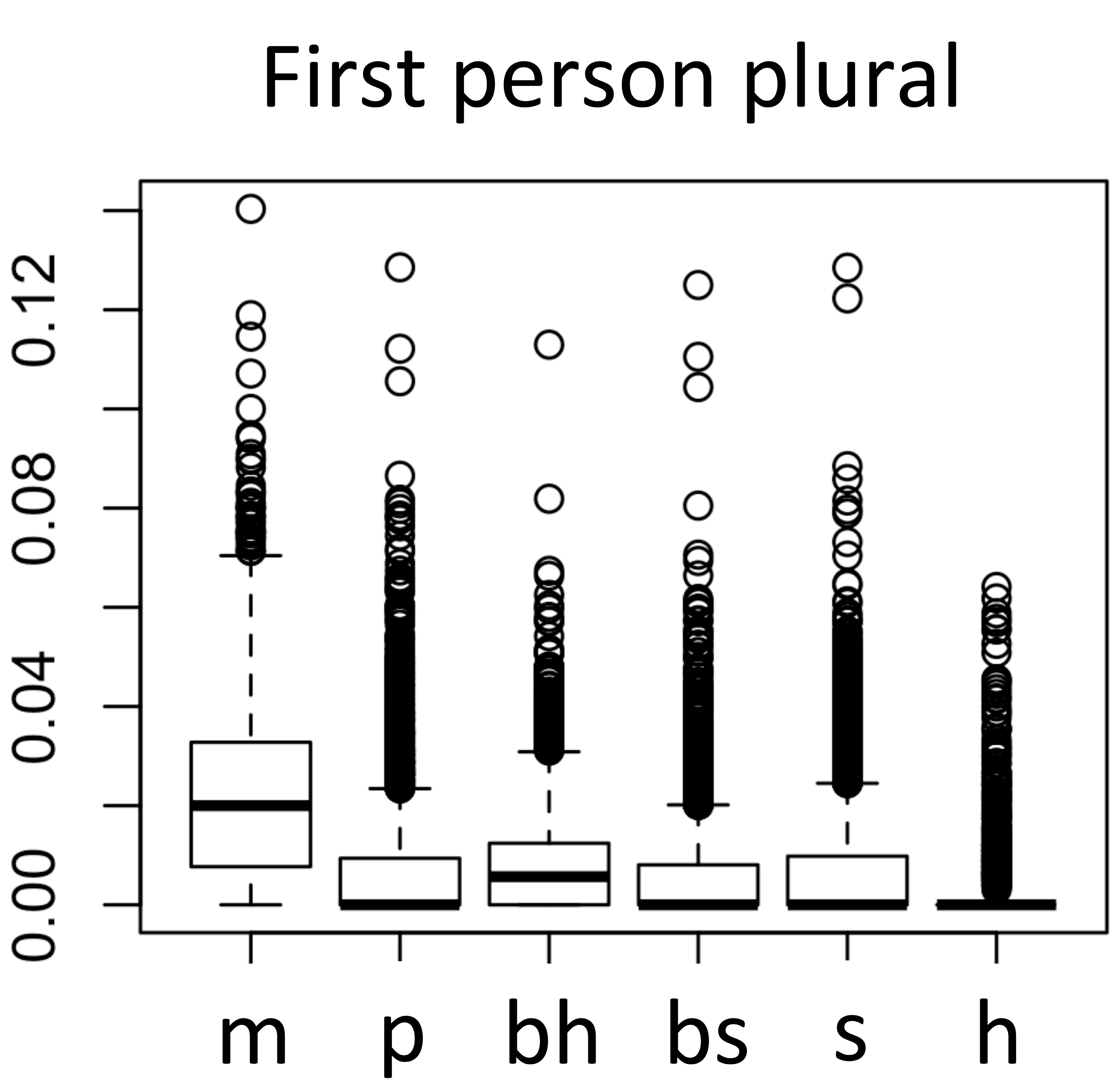

Supplement: Figure S1 — Frequency of first person plural pronouns in abstracts, by scientific domain. Domains are attributed based on journal, following the classifications of Essential Science Indicators and Arts & Humanities Science Citation Index: m = mathematics; p = physical sciences (Space Science + Physics + Chemistry); bh = hard-biological disciplines (Molecular Biology + Biology & Biochemistry); bs = soft-biological disciplines (Plant and Animal Sciences + Environment/Ecology); s = social sciences (Psychiatry/Psychology + Economics & Business + Social Sciences, general); h = Humanities. [Data sourced from Thomson Reuters Web of Knowledge]. (CORR) [file pone.0066938.s001.corr]

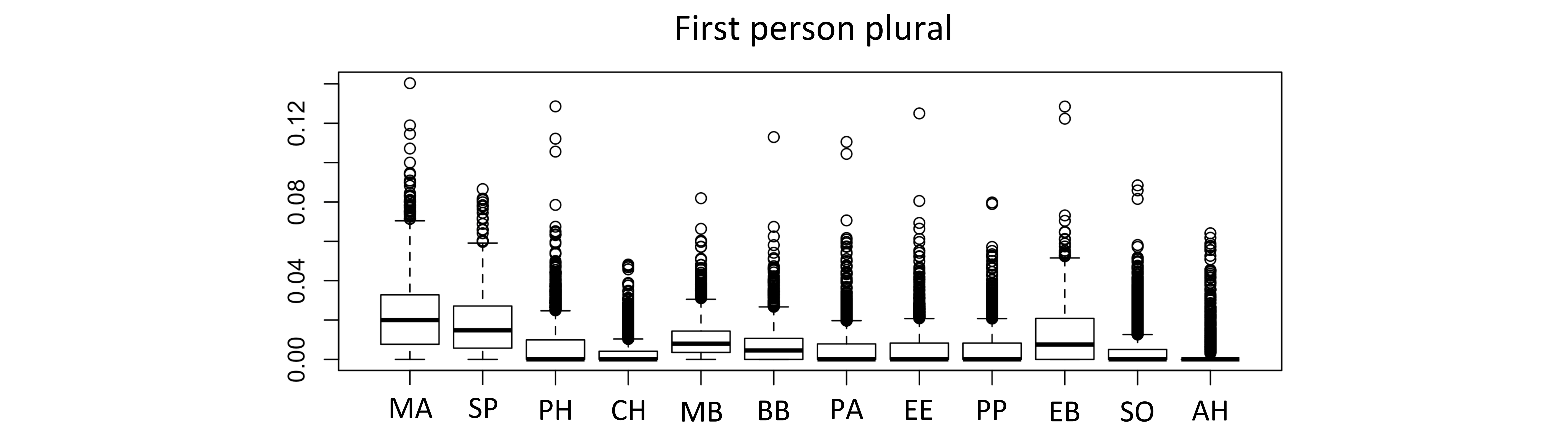

Supplement: Figure S2 — Frequency of first person plural pronouns in abstracts, by scientific domain. Domains are attributed based on journal, following the classifications of Essential Science Indicators and Arts & Humanities Science Citation Index: m = mathematics; p = physical sciences (Space Science + Physics + Chemistry); bh = hard-biological disciplines (Molecular Biology + Biology & Biochemistry); bs = soft-biological disciplines (Plant and Animal Sciences + Environment/Ecology); s = = social sciences (Psychiatry/Psychology + Economics & Business + Social Sciences, general); h = Humanities. [Data sourced from Thomson Reuters Web of Knowledge]. (CORR) [file pone.0066938.s002.corr]
