# Supplementary material for: Bibliometric Evidence for a Hierarchy of the Sciences
Source: PLoS One. 2013 Jun 26;8(6):e66938. doi: 10.1371/journal.pone.0066938 (PMC3694152; doi:10.1371/journal.pone.0066938)
Supplement: Table S1 — Main and extended test, individual parameters. (DOCX) [file pone.0066938.s003.docx]

**Table S1: Main and extended test, individual parameters**

|  | extended test | | | | |
| --- | --- | --- | --- | --- | --- |
|  |  | main test | | |  |
| predictor | m | bh | bs | s | h |
| ln(n. authors) | -0.667±0.016 | 0.224±0.009 | 0.043±0.009 | -0.375±0.009 | -1.914±0.042 |
| sqrt(n. references) | -0.187±0.008 | 0.138±0.006 | 0.152±0.005 | 0.159±0.005 | -0.275±0.009 |
| Price's index* | -0.430±0.022 | 0.005±0.015 | -0.347±0.015 | -0.288±0.014 | -0.533±0.028 |
| sqrt(Shannon diversity of sources)* | 0.451±0.085 | 2.550±0.064 | 3.068±0.061 | 4.209±0.059 | 0.952±0.096 |
| proportion of cited monographs* | 0.705±0.034 | 0.164±0.030 | 1.235±0.026 | 1.582±0.025 | 1.761±0.021 |
| ln(1+n. pages) | 0.626±0.012 | 0.168±0.009 | 0.276±0.009 | 0.569±0.008 | 0.066±0.014 |
| ln(relative title length)** | -0.991±0.015 | -0.058±0.013 | -0.123±0.012 | -0.682±0.012 | -0.305±0.017 |
| 1^st^ pers. singular *** | -0.068±0.245 | -0.474±0.242 | -0.697±0.212 | -1.127±0.204 | 1.033±0.197 |
| 1^st^ pers. plural*** | 0.012±0.049 | 0.653±0.116 | 0.960±0.123 | 0.505±0.053 | -0.02±0.086 |
| ln(1+sharing degree)**** | -0.133±0.036 | 0.894±0.027 | 1.052±0.026 | 1.133±0.025 | -1.684±0.041 |
| ln(1+sharing intensity)***** | 0.012±0.007 | -0.042±0.005 | -0.069±0.005 | -0.066±0.005 | -0.045±0.008 |

Table S1: Regression estimates, detailing those plotted in Figure 4. Physical sciences are the reference category; m=mathematics; bh= hard-biological disciplines (Molecular Biology + Biology & Biochemistry); bs= soft-biological disciplines (Plant and Animal Sciences + Environment/Ecology); s= social sciences (Psychiatry/Psychology + Economics & Business + Social Sciences, general), h=humanities. *weighted by sqrt(n. references); **weighted by log(1+n. pages); ***interaction term with binary dummy variable single-authored (-1) vs. multi-authored (+1), values reported are interaction terms in hierarchically well-formulated model, weighted by number of words in the abstract; ****weighted by sqrt.(n. references),; *****weighted by sqrt.(n. references), in model controlling for log(1+sharing degree). [Data sourced from Thomson Reuters Web of Knowledge]
